# Incorporating Direct Rapid Immunohistochemical Testing into Large-Scale Wildlife Rabies Surveillance

**DOI:** 10.3390/tropicalmed2030021

**Published:** 2017-06-30

**Authors:** Kevin Middel, Christine Fehlner-Gardiner, Natalie Pulham, Tore Buchanan

**Affiliations:** 1 Ontario Ministry of Natural Resources and Forestry, Wildlife Research and Monitoring Section, Peterborough, ON K9L 0G2, Canada; kevin.middel@ontario.ca (K.M.); natalie.pulham@ontario.ca (N.P.); 2 Canadian Food Inspection Agency, Centre of Expertise for Rabies, Ottawa, ON K2H 8P9, Canada; Christine.Fehlner-Gardiner@inspection.gc.ca

**Keywords:** dRIT, Ontario, rabies, surveillance

## Abstract

Following an incursion of the mid-Atlantic raccoon variant of the rabies virus into southern Ontario, Canada, in late 2015, the direct rapid immunohistochemical test for rabies (dRIT) was employed on a large scale to establish the outbreak perimeter and to diagnose specific cases to inform rabies control management actions. In a 17-month period, 5800 wildlife carcasses were tested using the dRIT, of which 307 were identified as rabid. When compared with the gold standard fluorescent antibody test (FAT), the dRIT was found to have a sensitivity of 100% and a specificity of 98.2%. Positive and negative test agreement was shown to be 98.3% and 99.1%, respectively, with an overall test agreement of 98.8%. The average cost to test a sample was $3.13 CAD for materials, and hands-on technical time to complete the test is estimated at 0.55 h. The dRIT procedure was found to be accurate, fast, inexpensive, easy to learn and perform, and an excellent tool for monitoring the progression of a wildlife rabies incursion.

## 1. Introduction

In December 2015 a raccoon (*Procyon lotor*) from Hamilton, Ontario, Canada, was diagnosed with rabies caused by the mid-Atlantic raccoon virus variant, the first case of rabies caused by this virus variant to occur in the province in 10 years. To assess the extent of this rabies outbreak and to direct rabies management actions, large-scale testing of raccoons and striped skunks (*Mephitis mephitis*) collected within a 50-km radius of diagnosed cases was undertaken. Animals were primarily collected by partnering agencies, such as municipal animal control agencies, humane societies and roads departments. Collection was focussed on sick-acting animals, animals found dead, or roadkill, and carcasses were kept frozen until they could be collected by Ministry of Natural Resources and Forestry (MNRF) staff. Frozen carcasses were collected weekly from partners and brought back to the MNRF laboratory for weekly testing using the direct rapid immunohistochemical test (dRIT). Samples diagnosed as rabies positive by dRIT were sent to the Canadian Food Inspection Agency (CFIA) for confirmation by fluorescent antibody test (FAT). Animals that had any previous contact with humans were not tested as part of this procedure. The dRIT test was developed by the Rabies and Poxvirus Section of the US Centers for Disease Control and Prevention (CDC), and was first evaluated in the field in Tanzania with very promising results [1]. The test was developed to provide a diagnostic methodology for quickly and inexpensively testing for rabies virus without the need for specialized equipment or facilities, making rabies testing more widely available worldwide, and has since been shown in many studies to be an effective tool for rabies surveillance [2]. The dRIT procedure was initiated in the rabies surveillance programs of the provinces of Québec [3] and Ontario in 2010. Until the most recent outbreak, the MNRF typically tested 10–40 animals annually, with four staff having been trained in dRIT procedures by CDC dRIT experts. Since raccoon rabies detection in December 2015, the dRIT surveillance program in Ontario has increased exponentially, and is currently the largest such surveillance program in Canada, testing anywhere from 23–258 (mean = 82.2) animals per week.

## 2. Materials and Methods

Test procedures: Brain tissue was collected by inserting a modified 3 mL or 1 mL syringe through the foramen magnum of animals and removing 1–2 mL of brain matter for touch impressions to slides for subsequent dRIT. dRIT was performed according to the CDC standard operating procedure as described previously [4], using a cocktail of two anti-nucleoprotein, biotinylated monoclonal antibodies (502 and 802) sourced from The Wistar Institute (Philadelphia, PA, USA). FAT was performed as described previously [5], using the same brain tissue samples collected for the dRIT. A fluorescein isothiocyanate-conjugated polyclonal antibody (goat serum, raised against purified ERA (Evelyn-Rokitnicki-Abelseth) rabies virus ribonucleoprotein), prepared in-house at the CFIA Centre of Expertise for Rabies (Ottawa, Canada), was used in the FAT.

Test comparison: Determination of dRIT sensitivity, specificity, and test agreement with FAT were estimated using an online calculator (EpiTools; http://epitools.ausvet.com.au).

## 3. Results

### 3.1. Surveillance Samples

**Figure 1:**
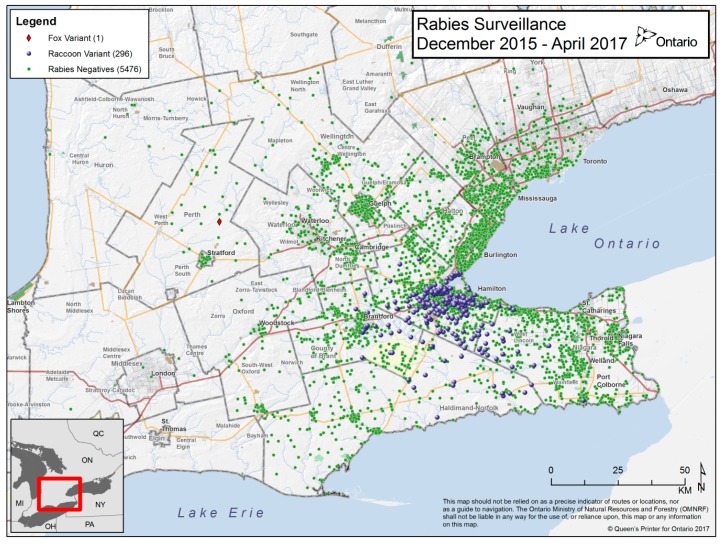
Ontario rabies surveillance map showing the negative (green) and positive (red and blue symbols) samples tested by dRIT.

In the 17-month period from December 2015 to April 2017, approximately 5800 mammalian wildlife carcasses were collected from southern Ontario and tested for rabies using the dRIT methodology (Figure 1). Species tested comprised primarily raccoons (80%) and striped skunks (17%). Other species tested included bat, feral cat (*Felis* spp.), coyote (*Canis latrans*), fisher (*Martes pennant*), red fox (*Vulpes vulpes*), groundhog (*Marmota monax*), muskrat (*Ondatra zibithecus*), opossum (*Didelphis virginiana*), cottontail rabbit (*Sylvilagus floridanus*), squirrel (*Sciurius* spp.), and weasel (*Mustela* spp.).

### 3.2. dRIT Performance in Comparison with FAT

All dRIT-positive samples were submitted to CFIA for confirmation using FAT. Of the 5800 wildlife samples tested by MNRF, 215 raccoons, 91 skunks and 1 red fox were diagnosed as being positive for rabies in the dRIT. When the same samples were tested by FAT, 205 of the raccoons, all the skunks and the fox were positive. Five hundred and fifty dRIT-negative samples were also tested in FAT. The samples were selected from random geographic locations from a time period between February and June 2016, and comprised 490 raccoons and 60 skunks. All of the dRIT-negative samples were also negative in the FAT. The dRIT had a sensitivity of 100% and specificity of 98.2% when compared with FAT (Table 1). Overall agreement between dRIT and FAT was high (98.8%, κ = 0.9744), with negative test agreement being slightly higher than positive test agreement (99.1% vs. 98.3%). On occasion, samples processed by dRIT were found to be inconclusive, typically due to poor sample conditions. Inconclusive samples were tested a second time to make a final dRIT diagnosis. In order to verify results of inconclusive samples where a second dRIT was conducted and was determined to be negative, 40 such samples were also evaluated by FAT. Of the 40 samples, 3 were untestable by FAT due to advanced sample decomposition; the remaining 37 samples had 100% diagnostic agreement between dRIT and FAT as being negative.

### 3.3. Cost

Through the collection and processing of approximately 5800 samples using dRIT procedures in the MNRF laboratory, an average cost of $3.13 CAD per sample for chemicals, reagents and laboratory consumables was estimated. In contrast, the materials cost for the FAT as performed at the CFIA is approximately $7.75 CAD per sample. Laboratory technical labour was comparable for the two tests, estimated at 0.55 h of hands-on time (i.e., not counting incubation periods), and thus labour costs are estimated to be similar, contingent upon local rates of remuneration. These estimates of cost do not include collection and transportation of samples, laboratory setup and maintenance, specimen disposal or administrative overhead, and were based on processing between 70 and 150 samples per week on average.

## 4. Discussion

The MNRF has responded to the most recent incursion of raccoon rabies by initiating an intensive surveillance program and testing over 5800 samples using the dRIT procedure. Since December 2015, 297 (96.4%) of the 308 cases of raccoon variant rabies diagnosed in Ontario have been diagnosed using MNRF dRIT surveillance, enabling the MNRF and partners to accurately and rapidly direct rabies control actions. Although dRIT and FAT diagnostic agreement was very high at 98.8%, it likely would have been closer to 100% were it not for a few early learning experiences with diagnostic procedures and processes. All 10 of the cases where samples were diagnosed positive by dRIT but negative by FAT were tested within the first three months of the outbreak. Additionally, during the same time period, samples that were inconclusive after the first dRIT test were sent directly to CFIA lab for additional testing by FAT. After receiving notification of these false positives, as well as dRIT-inconclusives that tested FAT-negative, two changes were implemented with regard to staff training and diagnostic processes. First, an expert in dRIT procedures was brought in to provide first-hand training to all staff performing the dRIT, to ensure that all test procedures were consistently done in accordance with the standardized protocol. Second, a new process, in which any sample with an inconclusive dRIT result was tested a second time before making a final dRIT diagnosis, was implemented. After implementing these two changes three months into the outbreak in February 2016, and in addition to staff gaining considerable experience with the dRIT test, agreement with FAT was 100% in the following 14 months and 4500 subsequent samples tested (of which 244 were positive). These data speak to the importance of thorough training and proficiency evaluation when implementing the dRIT, or any other diagnostic test for rabies. Based on these results, the dRIT has been found to be an excellent tool for monitoring the progression of a rabies incursion and will continue to be used for enhanced wildlife rabies surveillance within the province of Ontario. These data, along with those from many previous studies, also support the use of dRIT for improved rabies surveillance in regions where FAT cannot be practicably implemented.

## Figures and Tables

**Table 1:** Test comparison between fluorescent antibody test (FAT) and direct rapid immunohistochemical test (dRIT) for rabies.

	**FAT Positive**	**FAT Negative**	**Total**
dRIT positive	297	10	307
dRIT negative	0	550	550
Total	297	560	
	**Value**	**95% CI**	
dRIT Sensitivity	100.0%	98.77%–100.00%	
dRIT Specificity	98.21%	97.74%–99.14%	
Kappa	0.9744	0.9587–0.9902	
Negative agreement	0.9910	-	
Positive agreement	0.9834	-	
Overall agreement	0.9883	-	
